# Two Patients With Meigs' Syndrome and Elevated Serum CA-125: A Case Report

**DOI:** 10.7759/cureus.8927

**Published:** 2020-06-30

**Authors:** Javier Navarro-Esteva, Maria Laseca-Modrago, Octavio Arencibia-Sánchez

**Affiliations:** 1 Pulmonary Medicine, Hospital San Roque Maspalomas, Maspalomas, Las Palmas de GC, ESP; 2 Gynecologic Oncology, Complejo Hospitalario Universitario (C.H.U.) Insular-Materno Infantil, Las Palmas de GC, ESP

**Keywords:** meigs´ syndrome, ca-125, ovarian fibroma, pleural effusion

## Abstract

Patients with Meigs' syndrome and elevated serum CA-125 are not frequently reported. A 59-year-old woman and a 48-year-old woman sought help because of progressive shortness of breath caused by pleural effusion. The presence of a pelvic mass was noted in both the patients and was thought to be the cause of the effusion. Both patients had elevated serum CA-125, which raised the possibility of malignancy. After complete resection of the tumors, the pathologic reports confirmed a benign and a low-grade malignant ovarian neoplasia, respectively. We comment on the outcome and follow-up of these two cases and briefly review Meigs' syndrome.

## Introduction

The clinical presentation of a pelvic mass, ascites and pleural effusion is often associated with ovarian carcinoma. Furthermore, if blood and/or pleural fluid CA-125 are elevated, it raises suspicion for malignancy [1]. Herein we report two cases that presented with these features and turned out to have a benign and a low-grade malignant tumor, respectively.

## Case presentation

Case 1

**Figure 1:**
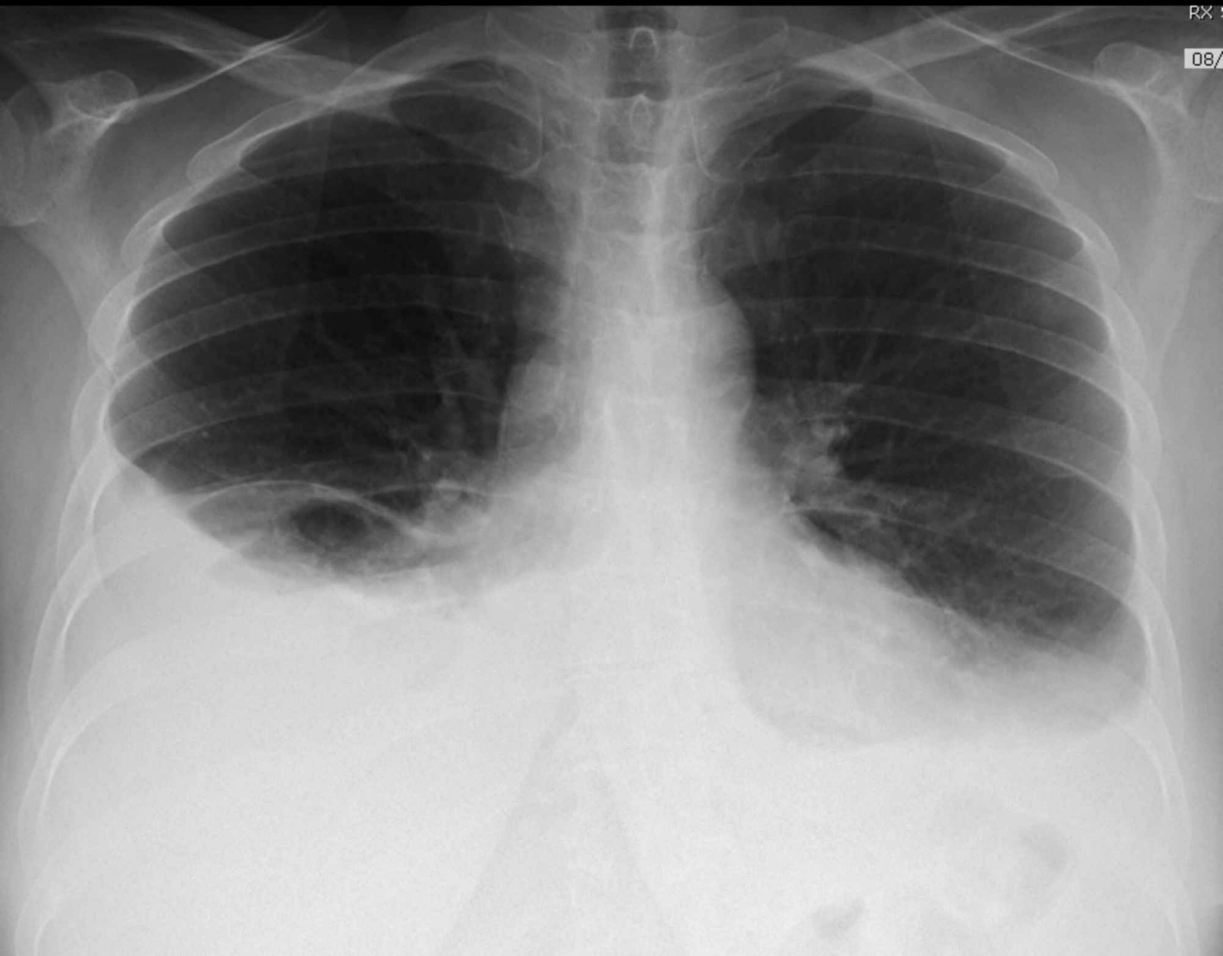
Chest X-ray. Bilateral pleural effusion distributed in a typical fashion, larger on the right side.

**Figure 2:**
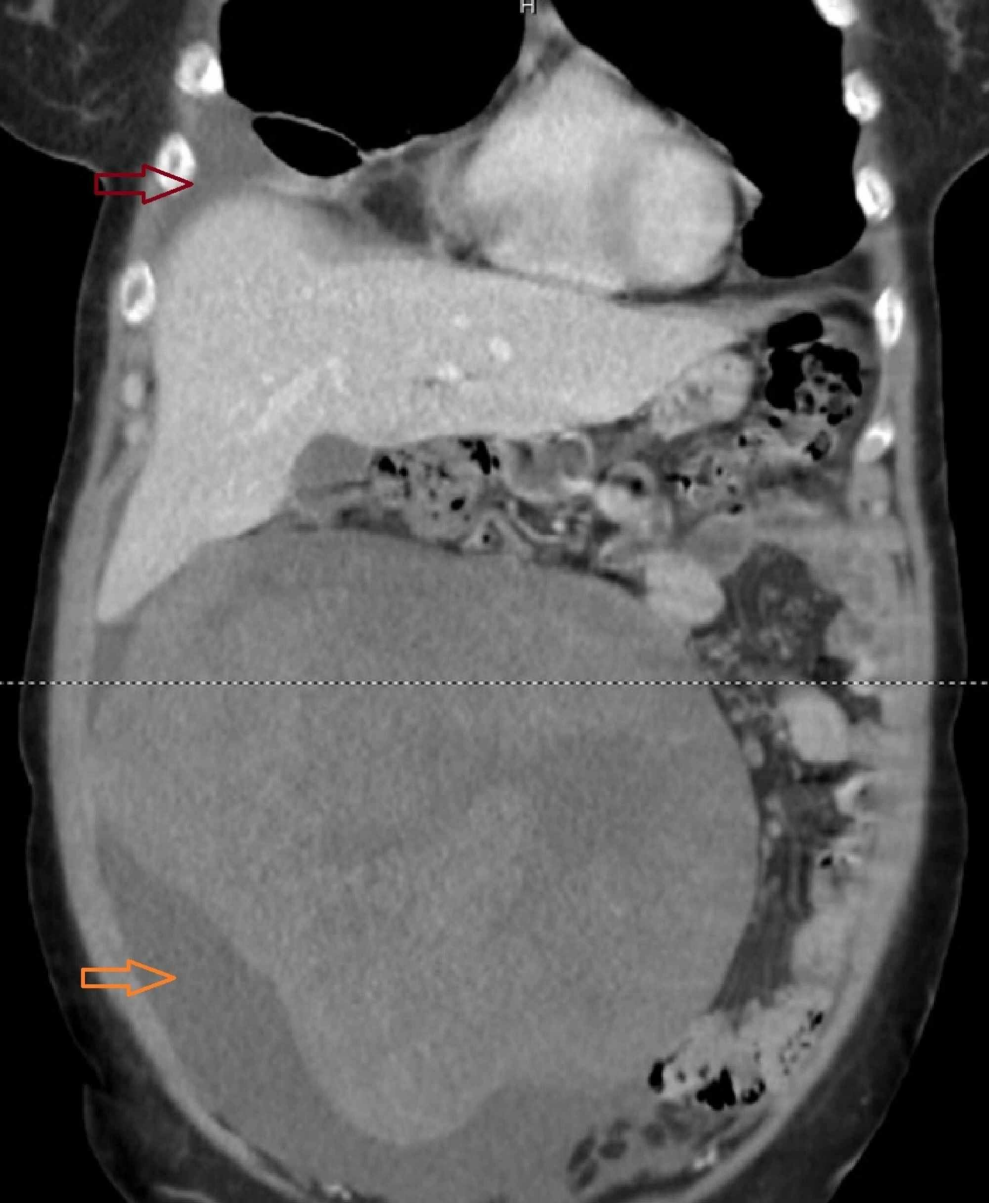
CT image, coronal view. A 21-cm abdominopelvic heterogeneous mass with clear borders and unclear origin – uterus versus ovary – is seen. Small amounts of peritoneal (orange arrow) and pleural fluid (red arrow) are observed. No lymphadenopathies were noted.

The first patient was a 59-year-old postmenopausal female who smoked 20 cigarettes per day (40 pack-year) with a known history of presumed uterine myoma three years before presentation but did not follow up with her physician. On this occasion, she presented to the emergency room (ER) complaining of progressively worsening dyspnea on exertion, dry cough, and discomfort over the right hemithorax elicited by deep inspiration. She denied weight loss or fever, but admitted to increased abdominal girth in the previous two years, neglecting to consult about it. On physical examination, she was slightly tachypneic with no accessory muscle use, room air SpO_2_ of 90%. Otherwise, the vital signs were normal. On auscultation, there were absent breath sounds and dullness to percussion over the lower half of the right hemithorax. The abdomen was globular and a firm, smooth, and slightly tender mass was palpated from the right hypochondrium to the pelvic area. No signs of ascites were evident. The chest X-ray and a representative CT image are presented (Figures 1, 2). Serum CA-125 was higher than 1000 IU/ml, HE-4 was within normal limits. The pleural fluid was a pale yellow exudate (fluid protein/serum protein ratio = 0.6) with pleural CA-125 level = 738 UI/ml. The cytology was negative for malignancy.

**Figure 3:**
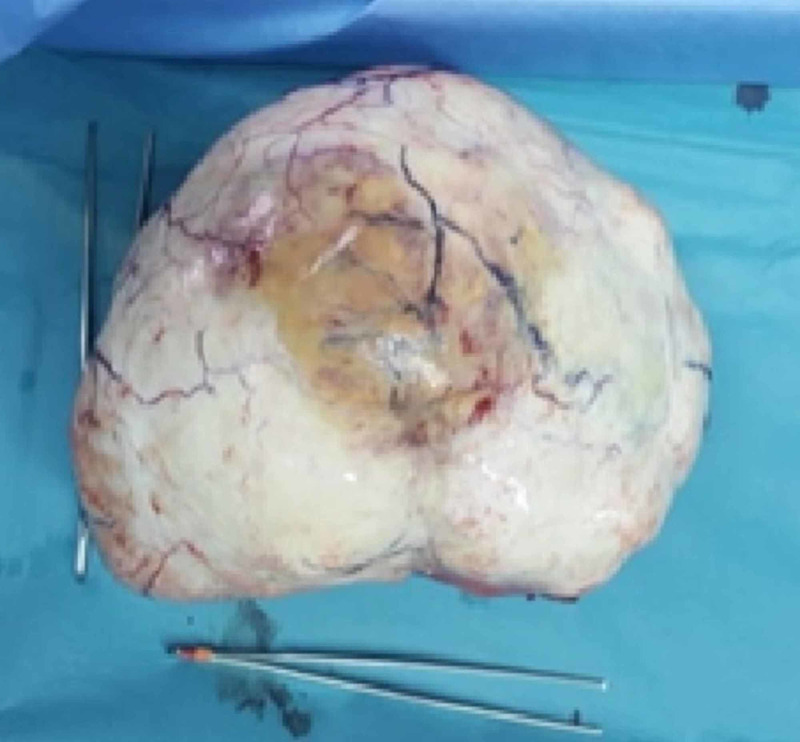
Right adnexal mass corresponding to an ovarian fibroma.

During the hospital admission, she reported increasing dyspnea due to accumulation of the pleural effusion that relieved after a therapeutic thoracentesis. A median laparotomy was performed. A 21-cm right adnexal solid mass with smooth and pearly appearance (Figure 3) and another 3-cm mass in the left ovary were noted and removed. The intraoperative pathologies of both masses were suggestive of ovarian fibroma, later confirmed by the examination of the whole piece. There were no other significant findings in the surgical exploration.

She was discharged two days after the surgery. On a follow-up visit three months later she reported no complaints, her lungs were clear on auscultation and the room air SpO_2_ was 98% with normal vital signs. She had stopped smoking. The patient refused to undergo a repeat chest X-ray.

Case 2

**Figure 4:**
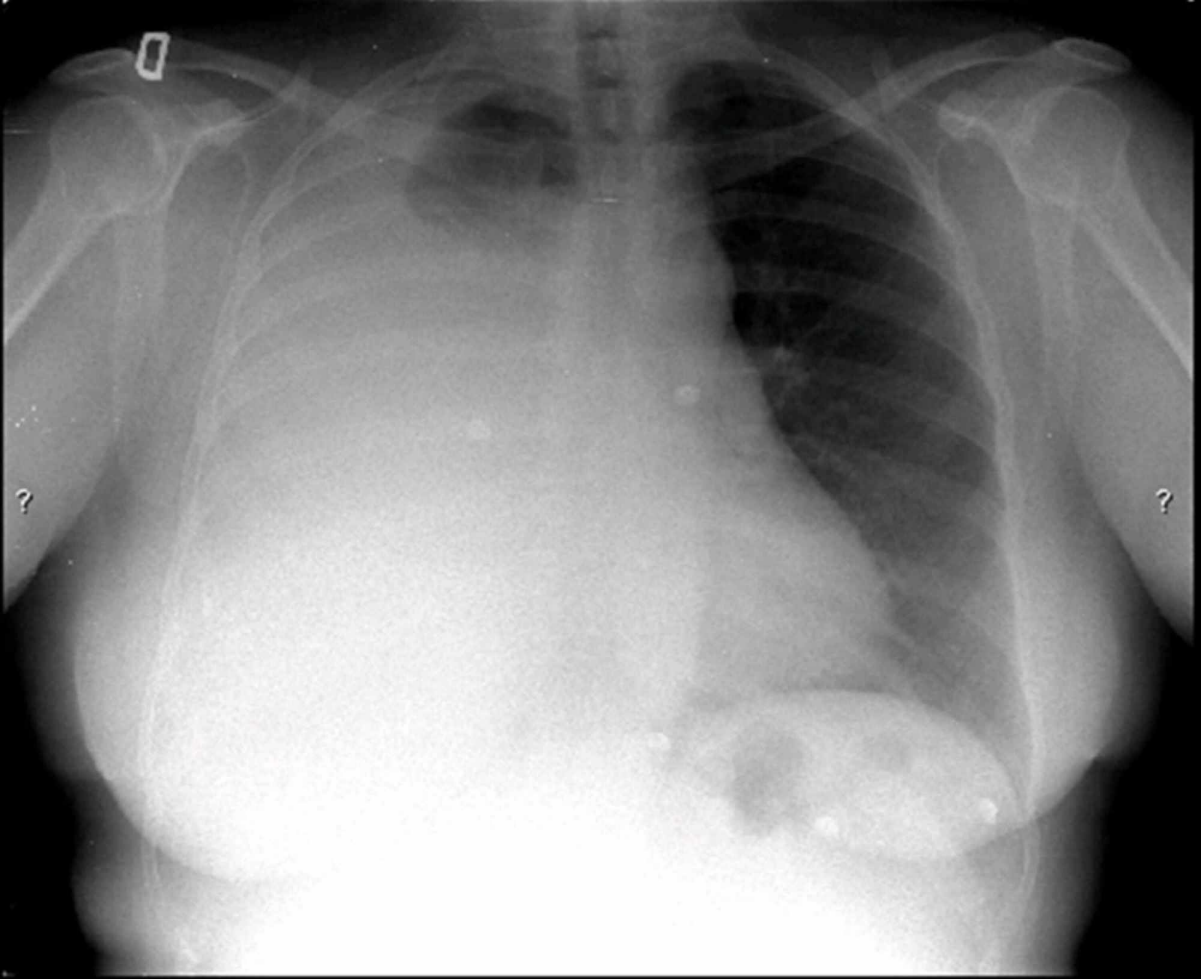
Chest X-ray on admission. A large right-sided pleural effusion is observed.

**Figure 5:**
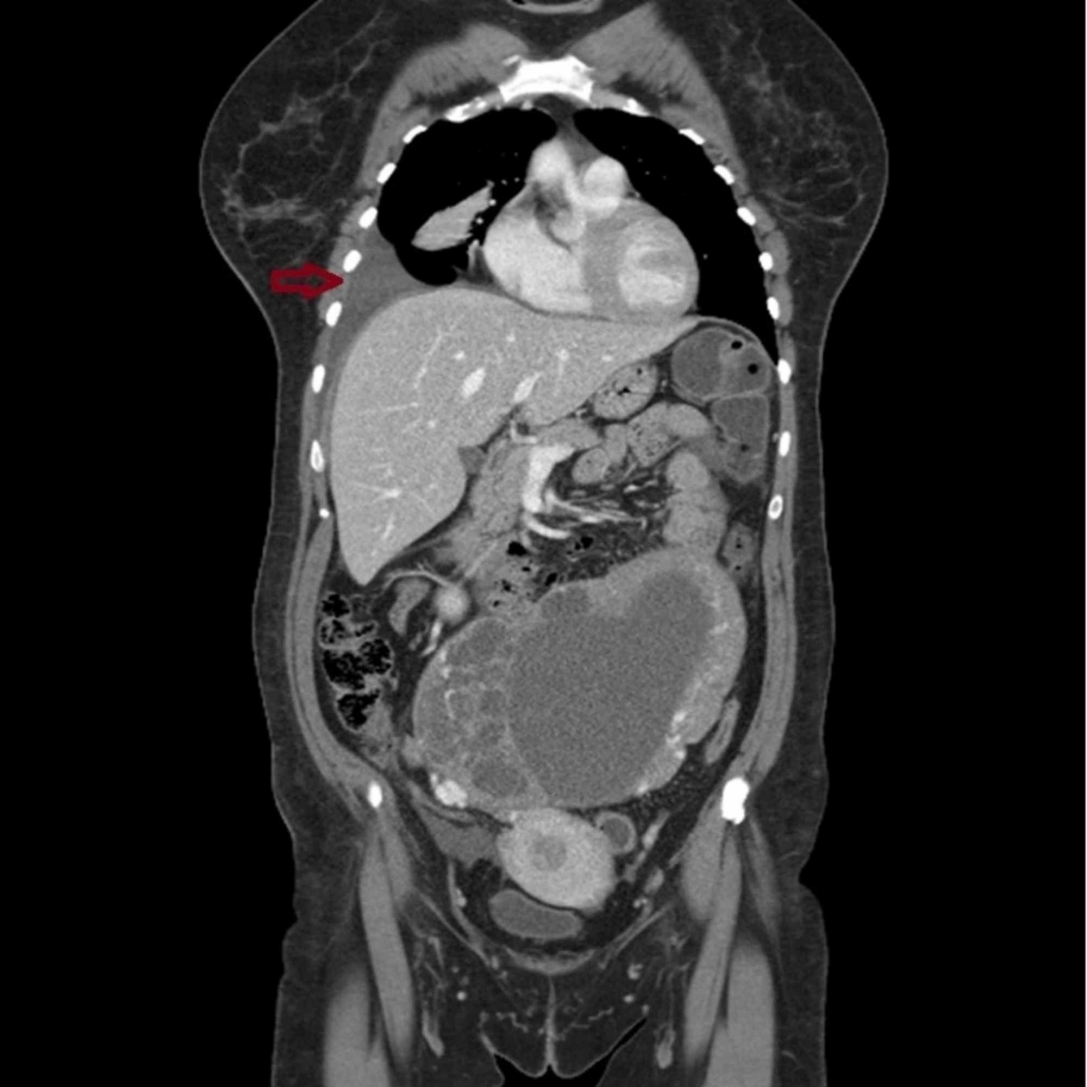
CT scan, coronal view. A heterogeneous pelvic mass of 17 cm diameter without lymphadenopathies was noted. The arrow points to the pleural effusion. A scanty amount of free peritoneal fluid was observed (not visible in this cut).

The second patient was a 48-year-old woman who complained of two weeks of progressive dyspnea associated with right-sided pleuritic chest pain and decreased appetite. Upon questioning, she revealed a progressive increase in abdominal girth and irregular menses in the previous year. The patient was slightly tachypneic without the use of accessory muscles, room air SpO_2_ was 95%. Other vital signs were normal. Breath sounds were absent on the right hemithorax and an abdominal mass with clear borders was palpated without clear signs of ascites. The chest X-ray and a representative CT image are shown (Figures 4, 5). The serum level of CA-125 was 526 U/ml. Due to the referred dyspnea, a diagnostic and therapeutic thoracentesis was performed, which was associated with the relief of the symptom. A pale yellow exudative pleural fluid (fluid protein/serum protein ratio = 0.7) was obtained. The cytology was negative for malignancy.

**Figure 6:**
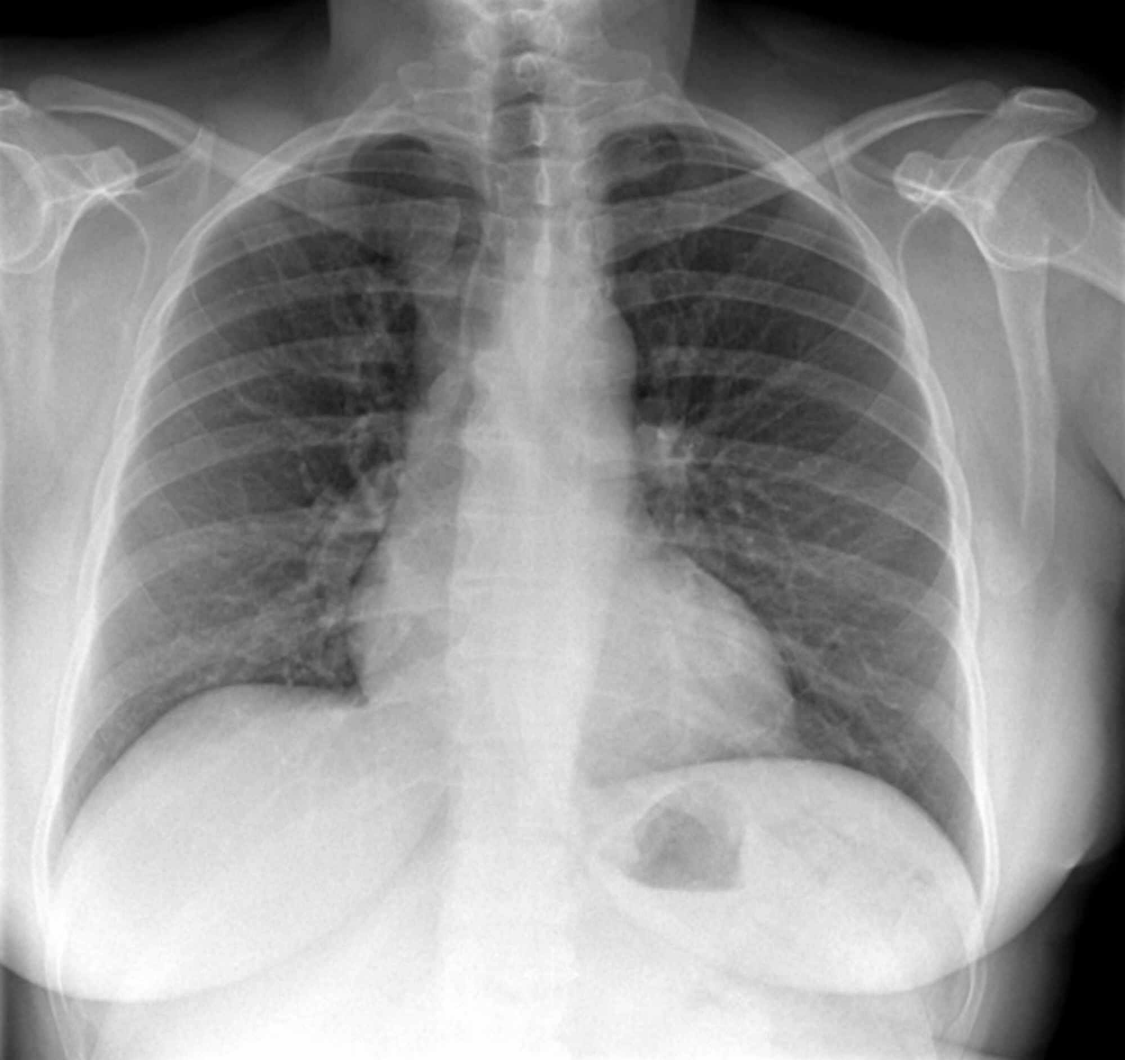
A follow-up chest X-ray three months after surgery shows complete disappearance of the pleural effusion.

The patient underwent a hysterectomy and bilateral oophorectomy. A solid-cystic irregularly surfaced and highly vascular mass that depended on the right adnexa was found. The appendix adhered to the mass. Peritoneal free fluid was present. Intraoperative biopsy of the lesion was informed as a granulosa cell tumor, stage IA, later confirmed after complete excision of the mass. Three months later, the patient reported no symptoms, had normal lung sounds, and a normal chest X-ray (Figure 6). She has been followed up for 10 years by the Gynecologic Oncology Department and has remained free of disease.

## Discussion

In the first case, the “myoma” suspected three years before presentation was an ovarian fibroma. Even though we could not obtain a postsurgical chest X-ray to document the disappearance of the pleural effusions, the patient meets otherwise all the criteria of a classic Meigs' or Demons-Meigs' syndrome. This syndrome was first reported in 1934, where removal of the fibroma resulted in the elimination of ascites and pleural effusion. Based on historical determinants, case reports, and review articles, four criteria should be met to classify a case as classic Meigs’ syndrome. These include: (a) benign fibroma (they account for up to 2% of resected ovarian neoplasms [2]) or the less common fibroma-like (thecoma, granulosa cell tumor, or Brenner tumor) ovarian tumors, (b) ascites, (c) pleural effusion, and (d) resolution of ascites and pleural effusion after removal of the tumor [1]. However, some authors also accept as Meigs' syndrome any case of low-grade malignant tumor associated with ascites and pleural effusion that disappear after surgical removal of the tumor [3]. The terminology may be somehow confusing, as Demons' syndrome refers to all benign genital tumors, Demons-Meigs' eponym is reserved for the description of ovarian fibromas and granulosa cell tumors, and Demons' pseudosyndrome includes all other entities [4]. Moreover, Pseudo-Meigs' syndrome, a clinical syndrome of pleural effusion, ascites, and an ovarian mass that is not a fibroma or fibroma-like mass/tumor, has been reported from a number of sources, particularly leiomyomas, struma ovarii, mucinous cystadenoma, teratoma, and malignancies that are metastatic to the ovary (particularly colorectal cancer) [5]. Imaging findings can help differentiate between benign and malignant tumors [6].

Meigs' syndrome presents more often in the fifth decade of life, and the cause of ascitic fluid appears to be a generalized secretion by the tumor itself. Moreover, the stimulation of the peritoneum's mesothelial cells by the release of vascular endothelial growth factor (VEGF), IL-1, IL-6, IL-8, and TNF-α from the tumor is reported as another cause of exudation. Only large tumors are frequently associated with ascites, which may not be detectable on physical examination, and the amount of ascites does not influence the amount of pleural effusion that develops. The genesis of the pleural effusion is similar to that of patients with hepatic hydrothorax, through pores in the diaphragm, and the similar characteristics between peritoneal and pleural fluid and the rapid reaccumulation of pleural fluid after evacuation supports this hypothesis. However, some authors believe that the pleural fluid arises from the transdiaphragmatic transfer of ascitic fluid by the lymphatic vessels, which seem to be larger on the right side, potentially explaining the finding of a right pleural effusion seen in approximately 70% of patients, being left-sided in 10%, and bilateral in 20%. Based on the protein levels, the effusion is usually an exudate with a low white blood cell count (fewer than 1000/mm^3^) and is occasionally bloody [3].

A CA-125 test is not accurate enough to use for ovarian cancer screening in general because many noncancerous conditions can increase the serum level, including normal conditions such as menstruation and pregnancy, and noncancerous conditions such as cirrhosis of the liver, uterine fibroids or endometriosis. Besides ovarian cancer, certain cancers may also cause an increased level of CA-125, including endometrial, peritoneal and fallopian tube cancers [7], while active smoking seems to decrease it [8]. Women with Meigs' syndrome may also have markedly elevated serum CA-125. Therefore, this finding should not be taken as an unequivocal sign of malignancy [1,3,9-12].

## Conclusions

The diagnosis of Meigs' syndrome should be entertained in all women with a pelvic mass, ascites and pleural effusion that present with a negative fluid cytology, and it is confirmed by the finding of a benign ovarian tumor and the nonrecurrence of peritoneal and pleural effusion once the mass is removed. It is important to be aware that, while awaiting for the pathology report, a high serum and/or fluid CA-125 does not compulsorily indicate malignancy.
